# 산후 2주 축약형 모유수유 적응 측정도구의 구성 타당도, 신뢰도와 측정 불변성

**DOI:** 10.4069/kjwhn.2020.11.10

**Published:** 2020-12-07

**Authors:** Sun-Hee Kim

**Affiliations:** College of Nursing, Daegu Catholic University, Daegu, Korea; 대구가톨릭대학교 간호대학

**Keywords:** Biological adaptation, Breast feeding, Instrumentation, Psychological adaptation, Validation study, 생리적 적응, 모유수유, 도구, 심리적 적응, 타당화 연구

## Abstract

**Purpose:**

This study was conducted to evaluate the construct validity, reliability, measurement invariance, and latent mean differences in the Breastfeeding Adaptation Scale-Short Form (BFAS-SF) for use with mothers at 2 weeks postpartum.

**Methods:**

This methodological study was designed to evaluate the validity, reliability, and measurement invariance of from 431 breastfeeding mothers. Confirmatory factor analysis and multi-group confirmatory factor analysis were conducted to assess the factor structure and the measurement invariance across employment status, delivery mode, parity, and previous breastfeeding experience, and the latent mean differences were then examined.

**Results:**

The goodness of fit of the six-factor model at 2 weeks postpartum was acceptable. Multi-group confirmatory factor analysis supported strict invariance of the BFAS-SF across employment status and delivery mode. Full configural invariance, full metric invariance, and partial scalar invariance across parity and full configural invariance and full metric invariance across previous breastfeeding experience were supported, respectively. The results for latent mean differences suggested that mothers who were employed showed significantly higher scores for breastfeeding confidence. Mothers who had a vaginal delivery showed significantly higher scores for sufficient breast milk and baby’s feeding capability. Multiparous mothers showed significantly higher scores for baby’s feeding capability and baby’s satisfaction with breastfeeding.

**Conclusion:**

The validity and reliability of the BFAS-SF at 2 weeks postpartum are acceptable. It can be used to compare mean scores of breastfeeding adaptation according to employment status, delivery mode, and parity.

## Introduction

### 연구 필요성

처음 모유수유를 하는 어머니와 아기는 산후 첫 주에 난생처음 겪는 신체, 심리, 환경적 변화로 인해 모유수유에도 많은 어려움과 도전을 겪는다[1]. 그리고 빠르면 산후 2주부터 안정적인 모유수유 적응이 되기 시작되며 산후 4–6주에는 대부분의 어머니는 모유수유가 정착된다[2,3]. 이 시기에 만약 모유수유 적응이 잘 이루어지지 않으면 어머니는 모성민감성이 낮아서 모아 애착 형성에 부정적인 영향을 주고, 정서와 스트레스에도 영향을 주어 모성행동에 부정적인 영향을 줄 수 있다[4], 또한 아기는 성장과 발달에 장애가 발생할 수 있다[5]. 그러므로 산후 가능한 한 조기에 모유수유 적응상의 문제를 발견하고 대응하는 것은 모아 모두에게 미칠 수 있는 부정적인 영향을 최소화할 수 있다. 산후 2주와 4주는 모유수유 적절성을 확인해야 하는 중요한 시기로 권고되고 있다[3]. 특히 산후 2주의 시점에서 모유수유 적응을 평가하는 것은 산후 산부인과와 소아과 외래 방문 시나 산후 추수 간호관리 시에 모성 스스로 도움을 요청하고, 간호사가 조기에 중재할 수 있는 적기이기 때문에 중요하다[2]. 따라서 산후 모유수유 적응을 조기에 확인하고 중재 마련의 발판을 확인하기 위해서 모유수유 적응이 되기 시작하는 산후 2주에 모유수유 적응을 평가하는 것이 도움이 될 수 있다.

모유수유 적응 측정도구[6]는 2009년에 산후 4주부터 사용하도록 개발되었다. 선행연구에서 산후 4주는 대부분 모유수유 적응이 되는 시점이기 때문에 이 시기의 모유수유 적응 평가는 필수적이고, 이후에도 모유수유 적응을 모성이 스스로 확인해보도록 하는 것에 목적을 두었다. 처음 개발 당시 모유수유 적응 측정도구는 27문항, 8요인으로 구성되었으며, 내용 타당도, 탐색적 요인 분석과 집단 비교를 통한 구성 타당도, 내적 일관성 신뢰도가 모두 수용 가능한 것으로 확인되었다. 이후 10년이 지난 2019년에 축약형 모유수유 적응 측정도구(Breastfeeding Adaptation Scale-Short Form, BFAS-SF) [2]가 보고되었다. BFAS-SF 역시 산후 4주 모유수유 중인 어머니에게 사용하는 것으로 16문항, 6요인으로 구성되었으며, 확인적 요인 분석을 통한 구성 타당도, 집단 간 측정 불변성, 내적 일관성 신뢰도를 검정하였다. 산후 2주와 4주의 기간에 따른 BFAS-SF의 종단적 측정 불변성 검정에서 형태 불변성과 부분 측정단위 불변성이 지지되어 두 기간의 BFAS-SF는 동일한 요인들을 측정하고, 2개 문항을 제외하고 14개의 각 문항이 해당 요인에 동일한 정도로 설명되고 있다는 점을 확인하였다[2]. 그러나 측정모형의 적합도가 좋지 않더라도 측정 불변성 검정은 가능하기 때문에[7] 종단적 측정 불변성 검정만으로 산후 2주 BFAS-SF의 구성 타당도를 확인하였다고 할 수 없고, 아직까지 산후 2주 BFAS-SF의 타당도와 신뢰도가 보고되지 않아 산후 2주의 타당한 사용을 위해서는 이를 확인할 필요가 있다.

최근 10년 이내의 연구에서 산후 4주 이내에 사용 가능한 측정도구로 축약된 모유수유 자기효능감 측정도구[8]와 모유수유 효과성 측정도구[9]가 보고되었다. 축약된 모유수유 자기효능감 측정도구[8]는 1개 요인, 14문항으로 구성된 것이며, 주로 모아의 수유 만족, 수유 방법, 수유 의지에 대한 문항으로 구성되어 있다. 이 도구는 BFAS-SF과 달리 모유수유를 하는 동안 아기와의 감정 교류, 충분한 젖의 양과 모유수유를 하기 위한 어머니의 노력인 젖의 양 유지에 대한 문항이 없다. 모유수유 효과성 측정도구[9]는 모성 만족, 젖 빨기, 젖의 양 확신, 아기 만족, 젖 물기, 수유 욕구, 수유 자세의 7개 하부영역으로 구성된 것으로, 직접 모유수유를 하는 동안의 모유수유의 효과성을 평가하는 도구이다. 이 도구 역시 BFAS-SF와 유사한 문항들이 있지만, 아기와의 감정 교류와 젖의 양 유지에 대한 문항은 없다. 즉, 축약된 모유수유 자기효능감 측정도구, 모유수유 효과성 측정도구, 그리고 BFAS-SF는 각각 평가 목적에 맞는 문항들로 구성된 것으로 유사한 문항들이 있지만, 각각의 도구는 다른 속성을 측정하는 문항들이 있어서 서로 대체 사용할 수 없다.

한편 모유수유 적응에 대한 선행연구에서 두 집단의 모유수유 적응 점수 비교 시 일관되지 않은 결과를 나타낸 집단의 특성이 보고되었으며[10-12], 그 집단의 특성은 직업, 출산력, 이전 모유수유 경험이었다. 또한 선행연구가 2개뿐이지만 이들 연구에서 질 분만과 제왕절개 분만 집단 간의 모유수유 적응은 차이가 없다고 나타났다[10,11]. 그러나 제왕절개 분만을 한 어머니는 출산 직후에 전신마취로 인해 질 분만한 어머니보다 옥시토신과 프로락틴 수치가 낮고[13], 산후 첫 모유수유 시도가 더 적기 때문에[14] 조기에 모유수유 자극이 적어 모유수유 적응이 지연될 수 있다. 이와 같이 기존의 연구에서 출산방법에 따른 모유수유 적응은 차이가 없다고 나타났지만, 생리적 원리에 기반한 논리와는 맞지 않기 때문에 출산방법에 따라 BFAS-SF의 측정 불변성을 확인하여 모유수유 적응을 정확하고 타당하게 비교할 필요가 있다. 즉, 집단 간에 어떤 측정변수의 점수를 정확하고 타당하게 비교하기 위해서는 측정 불변성이라는 전제가 담보되어야 하므로[15] 특성이 다른 집단 간의 모유수유 적응을 비교하기 위해서는 집단 간의 측정 불변성을 확인할 필요가 있다. 또한 집단 간 잠재 평균 비교를 통해 어떤 구성요인에서 두 집단 간의 평균에 차이가 있는지를 확인함으로써 집단에 따른 모유수유 적응의 특성을 확인할 뿐만 아니라 측정도구의 이론적 타당성에 대한 근거를 마련한다는 의미도 있다.

모유수유 부적응의 조기 감지와 대책 마련을 위해 산후 2주의 모유수유 적응 평가가 요구되고, 기존에 개발된 모유수유 적응 측정도구는 산후 4주부터 그 이후에 사용하도록 권장했으나 산후 4주 이내의 어머니에게도 적용한 선행연구들이 보고되고 있으므로[16,17] 산후 2주에 BFAS-SF의 적절한 사용을 위해 타당도와 신뢰도를 평가하는 것이 필요하다. 따라서 본 연구에서는 산후 2주에 적용하기 위한 BFAS-SF의 구성 타당도, 신뢰도, 측정 불변성을 확인하고, 더불어 잠재평균 비교를 하고자 한다.

### 연구 목적

본 연구에서는 산후 2주에 적용한 BFAS-SF의 구성 타당도, 신뢰도와 측정 불변성을 확인하는 것을 목적으로 하며, 구체적인 목적은 다음과 같다.

첫째, 산후 2주 BFAS-SF의 구성 타당도를 확인한다.

둘째, 산후 2주 BFAS-SF의 내적 일관성 신뢰도를 확인한다.

셋째, 산후 2주 BFAS-SF의 집단 간 측정 불변성을 확인한다.

넷째, 산후 2주 BFAS-SF의 집단 간 잠재평균의 차이를 확인한다.

## Methods

Ethics statement: This study was approved by the Institutional Review Board of Daegu Catholic University (CUIRB-20170079). Informed consent was obtained from the participants.

### 연구 설계

본 연구는 산후 2주 모유수유를 하는 어머니에게 적용한 BFAS-SF의 타당도와 신뢰도, 측정 불변성을 파악하기 위한 방법론적 연구이다.

### 연구 대상

본 연구의 대상자는 산후 2주인 14–20일에 직접 모유수유를 하고 있고, 모아가 모두 출산 후 질병으로 입원하지 않은 한국 여성이었다[2]. 산후 2주에 간접 모유수유만을 하고 있거나 모유수유를 일시적으로 중단하고 있는 자, 단유한 자는 제외하였다.

확인적 요인 분석을 위한 표본수를 산정하기 위해 Preacher와 Coffman [18]이 제시한 방법을 사용하였다. 즉, 확인적 요인 분석 모형을 확인하기 위해서는 영가설의 RMSEA .00과 대립가설의 RMSEA .05을 이용하는 Cronbach’s alpha .05와 검정력 .90을 만족시키며 설정한 모형의 자유도가 296일 때, 최소 109명이 필요하였다[2]. 그리고 두 집단 간의 측정 불변성을 검정하기 위해서는 집단마다 모형 적합도 확인을 위한 표본수가 각각 109명 이상 필요하므로 전체 218명 이상이 되어야 한다. 본 연구의 산후 2주의 목표 표본 수는 500명이었고, 444명이 설문지에 응답하였으며, 이 중에서 설문 응답이 불성실한 13부를 제외하여 최종 연구 대상은 431명이었다[2].

### 자료 수집

2017년 12월 15일부터 2018년 5월 17일까지 자료 수집을 하였다. 산후 2주의 모유수유 어머니를 대상으로 서울, 대구, 광주, 부산, 경상도에 위치한 10개의 산후조리원에서 편의 표집하였다. 산후조리원 원장에게 자료 수집에 대한 허락을 받은 후 각 산후조리원에서 근무하고 있는 자를 보조연구원으로 하였다[2]. 보조연구원은 연구 홍보물을 게시하여 자료 수집에 자발적으로 참여하기를 원하는 자에게 직접 연구에 대한 설명을 한 후에 서면 동의서를 받고 설문지를 배부 및 수거하였다[2]. 자료 수집에 참여한 자에게는 설문조사 직후 소정의 답례품을 제공하였다.

### 연구 도구

#### BFAS-SF

BFAS-SF는 6요인, 16문항으로 아기와의 감정 교류(4문항), 수유 자신감(3문항), 충분한 젖의 양(3문항), 아기의 수유 능력(2문항), 아기의 모유수유 만족(2문항), 젖의 양 유지(2문항)로 구성되었다[2]. 문항은 1점(전혀 그렇지 않다)에서 5점(매우 그렇다)의 5점 Likert 척도로 평가하였다. 하부영역과 전체 문항은 1–5점의 평균 평점을 산정하였고, 점수가 높을수록 모유수유 적응이 높음을 의미한다. 산후 4주의 BFAS-SF의 Cronbach’s alpha는 .88이었다[2].

### 자료 분석

본 연구는 IBM SPSS ver. 25.0 (IBM Corp., Armonk, NY, USA)을 사용하였다. 연구 대상자의 인구학적 특성은 빈도, 백분율로 분석하였고, 측정도구의 문항별 평균, 표준편차, 왜도, 첨도를 구하였다. 내적 일관성 신뢰도는 Cronbach’s α coefficient와 95% 신뢰구간으로 분석하였다.

확인적 요인 분석은 AMOS ver. 25.0 (IBM Corp., Armonk, NY, USA)를 사용하였다. 확인적 요인 분석을 통해 요인의 구성 타당도는 수렴 타당도, 판별 타당도로 평가하였다. BFAS-SF의 요인 구성 타당도 확인을 위한 수렴 타당도는 관찰 변수의 표준화 회귀계수, 개념 신뢰도, 평균 분산추출(average variance extracted, AVE)이었으며, 판별 타당도는 잠재변수 간 상관계수(Φ), 잠재변수의 AVE와 잠재변수 간 상관계수의 제곱(Φ^2^) 간의 크기 비교법을 사용하였다. 수렴 타당도 기준은 표준화 회귀계수 .50 이상, 개념 신뢰도 .70 이상, AVE .50 이상이었으며, 판별 타당도 기준은 요인 간 상관계수 .80 이하, 잠재변수의 AVE가 잠재변수 간 상관계수의 제곱 값보다 클 경우를 타당한 것으로 하였다[15]. 또한 모형 판별을 위한 모형의 적합도는 비교 적합지수(comparative fit index, CFI), 근사원소 평균자승 오차(root mean square error of approximation, RMSEA), 표준 원소 간 평균자승 잔차(standardized root mean residual, SRMR)를 사용하였다. 모형 적합도의 판별 기준은 CFI .900 이상, RMSEA .060 이하, SRMR .080 이하로 하였다[15].

BFAS-SF으로 두 집단 간의 측정 불변성은 1단계 형태 불변성(configural invariance), 2단계 측정단위 불변성(metric invariance), 3단계 절편 불변성(scalar invariance), 4단계 요인의 분산(공분산) 불변성(factor variance and covariance invariance), 5단계 오차의 분산 불변성(error variance invariance)을 확인하였다[15]. 각 단계에서 측정 불변성이 기각된 경우, 부분 측정 불변성을 확인하였다. 그리고 집단 간 잠재평균 비교는 절편 불변성을 전제로 하므로[15] 절편 불변성이 확보된 경우 집단 간 잠재평균을 비교하였다.

본 연구에서 측정 불변성 확인은 Chen [19]의 제안에 따라 주기준으로 CFI의 변화량(ΔCFI), 부기준으로 RMSEA의 변화량(ΔRMSEA), SRMR의 변화량(ΔSRMR)을 사용하였고, χ^2^의 차이검정은 표본 크기에 매우 민감하여 사용하지 않았다. 본 연구와 같이 전체 사례수가 300개보다 크고 직업 유무, 출산방법에 따른 두 집단의 표본수가 균등한 경우, 불변성 기준은 요인 부하량 검정에서 ΔCFI≤–.010, ΔRMSEA≤.015, ΔSRMR≤.030을, 절편과 측정오차 검정에서 ΔCFI≤–.010, ΔRMSEA≤.015, ΔSRMR≤.010를 사용하였다. 그리고 본 연구의 출산력, 이전 모유수유 경험에 따른 두 집단의 표본 수는 불균등하였기 때문에 더 엄격한 기준을 사용하였다. 이 경우의 불변성 기준은 요인 부하량 검정에서 ΔCFI≤–.005, ΔRMSEA≤.010, ΔSRMR≤.025를, 절편과 측정오차 검정에서 ΔCFI≤–.005, ΔRMSEA≤.010, ΔSRMR≤.005를 사용하였다.

## Results

### 대상자의 특성

본 대상자는 대부분 30대(75.3%)였으며, 학사 및 준학사 학위의 교육(82.4%)을 받았고, 가계 수입은 과반수 이상이 중간 정도(59.2%)였다. 대상자 중 직업이 있는 경우가 221명(51.3%)이었으며, 출산방법은 질 분만 223명(51.7%), 제왕절개 분만 208명(48.3%)이었다. 출산력은 초산이 316명(73.3%), 경산이 115명(26.7%)이었고, 이전에 모유수유 경험이 없는 경우가 324명(75.2%)이었다. 현재 아기는 모두 건강한 상태였고, 자녀 수는 1명(73.3%)이 대부분이었다. 기타 상세한 인구사회학적 특성은 선행논문에 제시하였다[2].

### 산후 2주 BFAS-SF의 타당도

BFAS-SF의 확인적 요인분석 모형의 적합도는 CFI .947, RMSEA .055 (90% 신뢰구간, .045–.065), SRMR .048로 나타나서 표본 크기에 민감한 χ^2^=205.16 (*p*<.001, 자유도 89)을 제외하고 모두 적합하였다. 표준화 회귀계수가 문항 7번(.47)을 제외하고 모두 .56 이상이었고, 다중상관제곱도 문항 7번(.22)을 제외하고 모두 .32 이상이었고, 요인의 분산과 오차 분산 모두 유의하였다(Table 1).

BFAS-SF의 요인의 수렴 타당도를 검정한 결과, 각 요인의 개념 신뢰도는 요인 2 (.66)를 제외하고 모두 .75 이상이었고, AVE도 요인 2 (.41)를 제외하고 모두 .61 이상이었다(Table 1). 또한 요인의 판별 타당도를 검정한 결과, 요인 1과 요인 3 간의 상관계수(Φ)는 유의하지 않아 서로 다른 속성을 측정하는 것으로 나타났고(*p*=.085), 나머지 요인 간 상관계수가 .20에서 .61로 나타나 상관성은 있지만 서로 다른 속성을 측정하는 것으로 판단되었다. 그리고 모든 잠재변수의 AVE가 잠재변수 간 상관계수의 제곱(Φ^2^)보다 큰 것으로 나타나 판별 타당도가 확인되었다(Table 1).

### 산후 2주 BFAS-SF의 신뢰도

산후 2주 BFAS-SF의 Cronbach’s α (95% 신뢰구간)는 전체 문항이 .82 (.80–.85)이었고, 1요인 .78 (.74–.81), 2요인 .57 (.50–.64), 3요인 .73 (.68–.77), 4요인 .79 (.75–.83), 5요인 .78 (.73–.82), 6요인 .72 (.67–.77)였다(Table 2).

### 산후 2주 BFAS-SF의 측정 불변성

산후 2주 BFAS-SF의 측정 불변성을 직업(유, 무), 출산방법(질 분만, 제왕절개 분만), 출산력(초산, 경산), 이전 모유수유 경험(유, 무)에 따라서 분석한 결과는 다음과 같다(Table 3). 산후 2주에 직업(유, 무)와 출산방법(질 분만, 제왕절개 분만) 각각에 따른 두 그룹 간 BFAS-SF의 측정 불변성을 검정한 결과, 형태 불변성, 측정단위 불변성, 절편 불변성, 요인의 분산(공분산) 불변성, 측정오차 불변성까지 확보되었다. 출산력(초산, 경산)의 두 그룹 간 BFAS-SF의 측정 불변성을 검정한 결과, 형태 불변성, 측정단위 불변성, 부분 절편 불변성(문항 7번, 10번 제외)까지 확보되었다. 이전 모유수유 경험(유, 무)의 두 그룹 간 BFAS-SF의 측정 불변성을 검정한 결과, 형태 불변성, 측정단위 불변성까지 확보되었다.

### 산후 2주 BFAS-SF의 집단 간 잠재변수의 평균 차이

측정 불변성 검정에서 절편 불변성이 확보된 후에 집단 간 잠재변수의 평균 차이를 확인하였으며, 산후 2주 BFAS-SF의 집단 간 잠재변수의 평균차이는 직업(유, 무), 출산력(초산, 경산)과 출산방법(질 분만, 제왕절개 분만)에 따라 분석하였다(Table 4). 산후 2주에서 직업이 없는 어머니는 직업이 있는 어머니에 비해 모유수유 적응의 잠재변인 2 (수유 자신감)가 평균 0.16 정도 낮았고(*p*=.043), 잠재변인 1, 3, 4, 5는 유의한 평균값의 차이가 없었다. 또한 질 분만을 한 어머니는 제왕절개 분만을 한 어머니에 비해 모유수유 적응의 잠재변인 3 (충분한 젖의 양)이 평균 0.35 정도 높았고(*p*<.001), 잠재변인 4 (아기의 수유 능력)가 평균 0.23 정도 높았다(*p*=.012). 경산모는 초산모에 비해 모유수유 적응의 잠재변인 4 (아기의 수유 능력)가 평균 0.25 정도 높았고(*p*=.010), 잠재변인 5 (아기의 수유 만족)가 평균 0.24 정도 높았다(*p*=.010).

## Discussion

본 연구는 모유수유 적응에 대한 선행연구[2,6]와는 다르게 산후 2주를 모유수유 적응 과정의 중요한 초기 평가시기로 보고, 산후 2주 BFAS-SF의 적용 가능성을 확인하고자 타당도와 신뢰도를 검정하였다. 그 결과 BFAS-SF은 수용 가능한 타당도와 신뢰도를 보여 산후 2주 모유수유 어머니에게 적용 가능한 것으로 나타났다. 또한 선행연구 간에 모유수유 적응의 차이가 불일치하게 나타난 변수를 선정하여 집단 간 측정도구의 측정 불변성을 확인함으로써 집단 간 동일한 요인 종류와 문항, 요인 부하, 절편, 요인 분산과 측정오차 분산을 나타내는지 검정하였다. 그 결과 산후 2주 대상자의 특성에 맞게 직업, 출산력, 출산방법에서 상당히 달성하기 어려운 (부분)절편 불변성 또는 측정오차 불변성까지 확보되었다. 이는 직업, 출산력, 출산방법 집단에 상관없이 사용 가능하다는 교차 타당성이 검정된 것이며, 하부 영역의 평균점수 비교 역시 가능한 도구임을 확인하였다. 또한 이전 모유수유 경험에 따른 BFAS-SF의 측정 불변성 결과는 측정단위 불변성까지 확보되고 절편 불변성은 지지되지 않아 이론적 근거에 합당하고 이전 모유수유 경험에 따른 산후 2주의 특성을 잘 반영한 도구임을 확인하였다.

좀 더 구체적으로 살펴보면, 본 연구에서 산후 2주 BFAS-SF의 구성 타당도를 확인한 결과 수렴 타당도에서 2요인인 수유 자신감 요인의 개념 신뢰도와 AVE가 기준 값에 비해 약간 낮았지만 0.6–0.7 사이의 개념 신뢰도도 수용할 수 있기 때문에[15] 비교적 적절하다고 생각한다. 또한 본 연구 결과에서 제시한 바와 같이 2요인에 해당되는 문항 7번(젖 먹이는 방법에 대해서 잘 알고 있다)의 표준화 회귀계수가 기준점보다 약간 낮았지만 기준점이 문항 제거의 절대적 기준은 아니며, 수유방법에 대한 지식은 수유 자신감 요인에 중요한 문항이기 때문에[20] 제거하지 않았다. 또한 문항 7번의 경우 수유 자신감 요인에 대한 설명력이 약간 낮은 것은 산후 2주 한국의 산후조리원의 실태를 잘 반영한 것이라고 생각할 수 있다. 즉, 우리나라는 산모의 약 75%가 산후조리원을 평균 13일 정도 이용하고 있고 모자 동실을 하루에 4.2시간 동안만 이용하고 있어서[21] 모아가 산후 첫 1, 2주 동안 모유수유 패턴을 익히고 적응하는 데 제한적일 수밖에 없다. 그러므로 산후 2주의 시기는 모유수유를 처음 하는 어머니가 모유수유 방법을 능숙하게 하기엔 이른 시기이므로 수유방법에 대한 지식 자체보다는 이전 모유수유 경험이 수유 자신감에 영향을 주었을 가능성이 있다. 이는 본 연구 결과에서 이전 모유수유 경험 집단 간에 모유수유 적응의 절편 값이 유의하게 차이가 있어서 측정 불편성을 확보하지 못한 것으로도 설명할 수 있다. 또한 요인의 판별 타당도 검정에서도 모두 수용 가능한 수준으로 나타나, 요인들은 모유수유 적응이라는 같은 개념을 측정하지만 서로 다른 속성(요인)들로 구성되어 있는 도구임을 확인하였다.

본 연구에서 산후 2주 BFAS-SF의 전체 문항의 신뢰도는 수용 가능한 수준이었다. 이는 산후 4주 BFAS-SF의 전체 문항의 신뢰도와 유사한 결과[2]로 나타났다. 보편적으로 Cronbach’s alpha 값의 표준으로 .70 이상이면 수용 가능한 것으로 사용되고 있지만[22] 이 수준은 모든 측정도구에 절대적인 표준이 된다고 할 수 없다. 그리고 신뢰도를 해석할 때 측정하려고 하는 하부 영역의 타당성과 전체 문항의 총수를 고려해야 한다[22]. 따라서 본 연구에서 하부 영역의 Cronbach’s alpha는 비록 중간 수준 이상이지만 확인적 요인 분석 결과로 구성된 것으로써 측정하고자 하는 내용을 측정한다고 볼 수 있으며, 전체 27문항에서 16문항으로 축소되었음에도 불구하고 신뢰도가 감소하지 않고 적절한 수준의 신뢰도를 보였다.

본 연구는 선행연구에서 일치되지 않은 연구 결과들의 원인으로 도구측 원인을 파악함과 동시에 BFAS-SF의 교차 타당성 검정의 하나로 측정 불변성을 확인하였다. 산후 2주 어머니의 직업 유무 집단 간에는 BFAS-SF의 형태 불변성부터 측정오차 불변성까지 5단계 모두 측정 불변성이 확보되었다. 이는 산후 초기인 2주에 직업 유무와 상관없이 산후 휴식 상태를 반영한 타당한 결과라고 볼 수 있다. 즉, 직장 여성이라고 하더라도 산후 2주는 아직 산후 직장 복귀를 준비해야 하는 시기가 아니므로 시기적으로 직업이 모유수유 적응에 영향을 미치지 않아 모유수유 적응 측정도구의 특성(요인 구조, 요인 부하량, 절편, 요인 간 변량/공변량, 오차분산/공분산)이 두 집단 간에 동일하게 나타났을 것으로 판단된다. 산후 4주에 직장 여성은 직장 복귀를 준비하기 위해 젖의 양을 줄이려고 모유수유를 위한 음식 섭취를 덜 하기 때문에 부분 측정단위 불변성, 부분 절편 불변성 등이 나타났다고 해석한 선행연구[2]의 결과를 고려해 볼 때, 향후 연구에서 산후 2주의 직업 유무에 따른 모유수유 적응 비교 시 타당한 해석이 가능할 것으로 생각한다.

산후 2주에 출산방법 집단 간 BFAS-SF의 측정 불변성은 아주 엄격한 오차분산의 불변성까지 확보되었다. 따라서 산후 2주 시점에서 두 집단 간의 모유수유 적응의 측정 특성이 같다는 것은 이 시점에서는 어떤 출산방법인지가 중요한 것이 아니라고 해석할 수 있고, 동일한 것을 측정한다고 해석할 수 있다. 이 결과 역시 산후 2주의 특성이 반영된 것으로, 이 시기는 산후조리원에 있는 시기이거나 산후조리 문화로 인해 아기와 산모를 돌보는 조력자가 충분히 있기 때문에[21] 모유수유 적응 측정의 속성에서 차이가 미미했을 수도 있다. 따라서 본 연구의 측정 불변성 결과에 근거하여 향후 연구에서 두 집단 간에 모유수유 적응 점수 비교가 가능하다고 판단된다.

산후 2주 초산과 경산 집단 간에 BFAS-SF의 측정 불변성 역시 출산력에 따른 산후 2주의 특성을 타당하게 반영한 결과라고 볼 수 있다. 즉, 산후 2주에 초산과 경산 집단 간에 BFAS-SF의 형태 불변성, 측정단위 불변성이 확보되었고, 7번 문항(젖 먹이는 방법에 대해서 잘 알고 있다)과 10번 문항(젖 먹일 때가 되면 찌릿하게 젖이 도는 느낌이 든다)의 절편을 제외하고 나머지 문항들의 절편 불변성까지 확보되었다. 이는 선행연구[2]에서 초산모보다 경산모는 수유방법을 더 잘 알고 있기 때문에 본 연구에서 초산과 경산 집단 간에 7번 문항의 절편 값 차이가 크게 나타났을 것으로 생각된다. 또한 경산모는 초산모에 비해 유선 세포의 발달이 더 잘되기 때문에 유즙 분비량과 저장 용량이 더 많다[23]. 그리고 경산모는 이전에 모유수유를 했을 가능성이 있으므로 산후 2주에 초산모보다 신체 생리적인 수유에 더 빨리 적응하여 수유 시 젖 사출이 잘 되고 젖 사출의 감각을 더 잘 알아차렸을 것으로 볼 수 있다. 나머지 14개 문항의 경우 절편 불변성이 확인되었으므로 두 집단 간 평균 비교는 가능하되, 7번 문항이 포함된 수유 자신감 하부 요인과 10번 문항이 포함된 충분한 젖의 양 하부 요인의 평균 비교 시 해석에 주의를 요한다.

두 집단의 절편 불변성이 지지되어 잠재평균 비교를 한 결과를 구제척으로 살펴보면, 본 연구에서 산후 2주에 직업이 없는 어머니는 직업이 있는 어머니보다 모유수유 적응의 수유 자신감 하부 영역을 제외하고 다른 하부 영역의 잠재평균에 차이가 없었다. 이는 산후 2주는 어머니의 모유수유 적응이 시작되는 시점이고 직장 복귀를 고려할 시기가 아니기 때문에[2,3] 직업 유무와 상관없이 아기와의 감정 교류, 충분한 젖의 양, 아기의 수유 능력, 아기의 수유 만족, 젖의 양 유지 부분에서 별 차이가 없었다고 해석할 수 있다. 또한 산후 2주에 질 분만을 한 어머니는 제왕절개 분만을 한 어머니에 비해 모유수유 적응의 충분한 젖의 양, 아기의 수유 능력 하부 영역의 잠재평균이 유의하게 높았고, 다른 하부 영역의 잠재평균은 차이가 없었다. 제왕절개 분만을 한 어머니의 경우 산후 1시간 이내에 모유수유 시행과 빈도가 더 적어[14] 산후 초기 유즙 생성이 지연되며, 유즙 생성 2단계(stage II lactogenesis)인 산후 3–8일에 유즙 생성에 실패하면 산후 9일 이후 모유 분비량 유지가 어렵다[24]. 따라서 산후 2주 제왕절개 분만을 한 어머니는 질 분만을 한 어머니에 비해 젖의 양 생성이 충분하지 못했을 수 있고, 본 연구에서 모든 대상자의 아기는 건강한 상태였기 때문에 신체적 수유능력과 관련된 신경과 근육, 소화기계 발달에 차이가 없었을 것으로 생각된다. 또한 제왕절개 분만을 한 어머니가 젖의 양 부족으로 아기가 젖 빨기와 삼키기를 잘 못한다고 판단했을 가능성과 보충 수유로 인해 아기의 유두 혼동과 같은 문제를 경험했을 가능성[24]도 고려해볼 수 있다.

본 연구에서 산후 2주 출산력에 따라 아기의 수유능력 하부 영역과 아기의 수유 만족 하부 영역에서 잠재평균 차이가 있었다. 본 연구의 아기는 모두 건강한 아기였기 때문에 신체적인 수유능력은 비슷했을 것으로 추정되며, 이전 모유수유 경험으로 인해 유방과 유두 상태가 모유수유에 더 적절한 상태가 되어[23] 아기의 수유능력에 차이를 주었고 결론적으로 아기의 수유 만족에도 차이를 주었을 것으로 해석된다. 초산모는 경산모보다 함몰(편평)유두와 유두 통증, 불충분한 수유, 부적절한 젖의 양 등의 수유 문제가 더 많은데[25], 산후 2주에 수유 문제가 가장 심하기 때문에[26] 아기가 젖 물기와 빨기를 잘 하지 못하고 수유에 만족하지 못했을 것으로 생각된다.

이상과 같이 본 연구는 산후 2주 16문항의 BFAS-SF의 구성 타당도, 신뢰도, 측정 불변성을 검정하였다. 그 결과 산후 2주의 모유수유 어머니에게 적용 가능하고, 타당하고 신뢰할 수 있는 도구라는 것을 확인하였다. 특히 선행연구들 간에 불일치한 결과를 보였던 변수들을 선정한 후 집단간 측정 불변성 검정을 통해 직업, 출산방법, 출산력, 이전 모유수유 경험에 따른 두 집단 간의 측정 불변성(교차 타당성)을 확인하였고, 집단 간 모유수유 적응의 비교 타당성과 비교 시 고려할 점을 제시하였다. 또한 선행연구들 간에 불일치한 결과의 도구 측 원인을 파악하기 위해 측정 불변성 검정을 한 결과, 직업, 출산방법, 출산력, 이전 모유수유 경험에 따른 산후 2주 모유수유 적응 평가에서 응답반응에 대한 측정도구 자체의 문제는 배제할 수 있음을 확인하였다. 본 연구는 2019년 선행연구[2]의 후속 연구로서 산후 2주의 타당도와 신뢰도 평가 결과를 보고하였다. 본 연구 결과를 바탕으로 향후 임상에서 산후 2주의 어머니에게 BFAS-SF를 활발히 활용하길 기대한다.

본 연구는 자료 수집의 협조를 얻지 못하여 산후조리원을 이용하지 않는 산모에게 자료를 수집하지 못하였고 산후조리원을 이용한 산모만을 자료 수집하였기 때문에 연구 결과를 전체 산모에게 일반화하기 어렵다. 향후 연구에서는 측정도구의 일반화를 위해 산후조리원을 이용하지 않은 표본으로 교차 타당도를 검정할 필요가 있다. 또한 본 연구에서 확인하지 못한 준거 타당도 검정을 제언한다.

## Figures and Tables

**Table 1.** Convergent and discriminant validity of the Breastfeeding Adaptation Scale-Short Form at 2 weeks postpartum (N=431)

Factor	Observational variable	B	β	CR	SMC	Construct reliability	AVE	F1 (Φ)	F2 (Φ)	F3 (Φ)	F4 (Φ)	F5 (Φ)	F6 (Φ)
F1	Item 1	1.00	.58*		.34*	.90	.83	1	.58**	.11	.20**	.27**	.28**
	Item 2	2.37	.79*	11.64	.62*								
	Item 3	2.71	.83*	11.67	.69*								
	Item 4	2.50	.64*	9.93	.41*								
F2	Item 5	1.00	.56**		.32**	.66	.41		1	.43*	.48*	.48*	.40**
	Item 6	0.85	.72**	9.23	.52**								
	Item 7	0.57	.47*	6.80	.22*								
F3	Item 8	1.00	.81*		.66*	.75	.61			1	.35*	.61**	.35*
	Item 9	0.68	.70*	11.61	.49*								
	Item 10	0.64	.57*	10.29	.33*								
F4	Item 11	1.00	.77*		.59*	.80	.73				1	.60*	.29**
	Item 12	0.98	.87**	11.38	.75**								
F5	Item 13	1.00	.84**		.71**	.79	.71					1	.28*
	Item 14	0.93	.76*	13.17	.57*								
F6	Item 15	1.00	.72*		.51*	.84	.76						1
	Item 16	0.91	.81**	6.52	.66**								

AVE: Average variance extracted; CR: critical ratio; F1: emotional exchange with one’s baby; F2: breastfeeding confidence; F3: sufficient breast milk; F4: baby’s feeding capability; F5: baby's satisfaction with breastfeeding; F6: maintenance of breast milk volume; SMC: squared multiple correlation; β: standardized regression coefficient; Φ: correlation coefficient.

* *p*<.050,

** *p*<.010.

**Table 2.** Reliability of the Breastfeeding Adaptation Scale-Short Form at 2 weeks postpartum (N=431)

Factor	Item	Mean±SD	Skewness	Kurtosis	Cronbach’s α (95% CI)
F1	1. My baby looks so lovely when he or she is drinking breast milk.	4.82±0.38	–1.70	0.90	.78 (.74–.81)
	2. I feel the exchange of good emotions while breastfeeding my children.	4.49±0.67	–1.09	0.59	
	3. I am happy during breastfeeding.	4.35±0.72	–0.86	0.42	
	4. I seem to have become a true mother when breastfeeding.	4.18±0.87	–0.72	–0.33	
F2	5. I am going to breast feed for over 6 months.	3.82±1.14	–0.63	–0.65	.57 (.50–.64)
	6. I can endure breastfeeding even with difficulties.	3.81±0.76	–0.28	0.00	
	7. I know well about how to feed breast milk.	3.30±0.77	0.15	–0.01	
F3	8. My milk is sufficient for my baby’s intake.	3.30±1.08	0.09	–0.84	.73 (.68–.77)
	9. I have no problem feeding my baby because I have good nutrition.	3.66±0.85	–0.25	–0.20	
	10. My breasts feel full when it is time to feed my baby.	3.97±0.99	–0.84	0.05	
F4	11. My baby latches on my breast and sucks well.	3.62±1.04	–0.40	–0.57	.79 (.75–.83)
	12. My baby sucks milk with a regular rhythm and swallows it.	3.60±0.91	–0.28	–0.29	
F5	13. My baby is satisfied after breastfeeding.	3.35±0.94	–0.12	–0.35	.78 (.73–.82)
	14 My baby does not cry during or after breastfeeding.	3.41±0.97	–0.30	–0.49	
F6	15. I try to get enough rest and sleep for breastfeeding.	3.80±0.78	–0.45	0.30	.72 (.67–.77)
	16. I try to eat enough food and water for breastfeeding.	4.16±0.63	–0.30	0.16	
Total		3.85±0.84			.82 (.80–.85)

CI: Confidence interval; F1: emotional exchange with one’s baby; F2: breastfeeding confidence; F3: sufficient breast milk; F4: baby’s feeding capability; F5: baby's satisfaction with breastfeeding; F6: maintenance of breast milk volume.

**Table 3.** Measurement invariance of the Breastfeeding Adaptation Scale-Short Form at 2 weeks postpartum (N=431)

Variable	Invariance model	Model fit indices						Model comparison		
		χ^2^	df	NC	CFI	RMSEA	SRMR	ΔCFI	ΔRMSEA	ΔSRMR
Employment status	Configural	320.78*	178	1.80	.936	.043	.068			
	Metric	335.02*	188	1.78	.934	.043	.068	–.002	.000	<.001
	Scalar	364.83*	204	1.79	.928	.043	.069	–.006	.000	.001
	Factor variance/covariance	383.70*	225	1.71	.928	.041	.077	<.001	–.002	.008
	Error variance	421.87*	241	1.75	.918	.042	.078	–.010	.001	.001
Delivery mode	Configural	336.65*	178	1.89	.929	.046	.056			
	Metric	345.02*	188	1.84	.929	.044	.057	<.001	–.002	.001
	Scalar	372.76*	204	1.83	.924	.044	.057	–.005	.000	<.001
	Factor variance/covariance	408.82*	225	1.82	.917	.044	.066	–.007	.000	.009
	Error variance	432.68*	241	1.80	.914	.043	.067	–.003	–.001	.001
Parity	Configural	320.35*	178	1.80	.936	.043	.048			
	Metric	336.77*	188	1.79	.933	.043	.050	–.003	<.001	.002
	Scalar	410.47*	204	2.01	.907	.049	.050	–.026	.006	<.001
	Partial scalar^†^	362.70*	202	1.80	.928	.043	.051	–.005	.000	.001
Previous breastfeeding experience	Configural	318.71*	178	1.79	.936	.043	.045			
	Metric	331.07*	188	1.76	.935	.042	.047	–.001	–.001	.002

CFI: Comparative fit index; df: degree of freedom; NC: normed chi-square; RMSEA: root mean square error of approximation; SRMR: standardized root mean residual; Δ: difference of value.

* *p*<.001.

† All items except item 7 and 10.

**Table 4.** Mean between-group differences in latent variables at 2 weeks postpartum (N=431)

Variable	Factor	Mean	SE	CR	*p*
Employment status^†^	F1	–0.05	.02	–1.92	.055
	F2	–0.16	.08	–2.02	.043
	F3	–0.10	.10	–0.98	.327
	F4	0.01	.08	0.14	.886
	F5	–0.05	.09	–0.56	.577
	F6	–0.05	.06	–0.81	.420
Delivery mode^†^	F1	0.00	.02	–0.17	.864
	F2	0.09	.08	1.19	.234
	F3	0.35	.10	3.60	<.001
	F4	0.23	.09	2.52	.012
	F5	0.09	.09	1.02	.308
	F6	0.09	.07	1.36	.174
Parity^†^	F1	0.00	.03	0.09	.930
	F2	0.12	.09	1.44	.151
	F3	0.11	.11	0.96	.340
	F4	0.25	.10	2.59	.010
	F5	0.24	.10	2.57	.010
	F6	–0.07	.07	–0.91	.364

CR: Critical ratio; SE: standard error; F1: emotional exchange with one’s baby; F2: breastfeeding confidence; F3: sufficient breast milk; F4: baby’s feeding capability; F5: baby’s feeding satisfaction; F6: maintenance of breast milk volume.

† Dummy variable references were employment (yes), delivery mode (cesarean section), and primiparity (yes).
